# Increased risk of coronary heart disease among patients with primary Sjögren’s syndrome: a nationwide population-based cohort study

**DOI:** 10.1038/s41598-018-19580-y

**Published:** 2018-02-02

**Authors:** Xue-Fen Wu, Jing-Yang Huang, Jeng-Yuan Chiou, Huang-Hsi Chen, James Cheng-Chung Wei, Ling-Li Dong

**Affiliations:** 1 Department of Rheumatology and Immunology, Tongji Hospital, Tongji Medical College, Huazhong University of Science and Technology, Wuhan, Hubei China; 2 Department of Medical Research, Chung Shan Medical University Hospital, Taichung, Taiwan; 3 School of Health Policy and Management, Chung Shan Medical University, Taichung, Taiwan; 4 Institute of Medicine, Chung Shan Medical University, Taichung, Taiwan; 5 Department of Medicine, Chung Shan Medical University Hospital, Taichung, Taiwan; 6 Graduate Institute of Integrated Medicine, China Medical University, Taichung, Taiwan

## Abstract

To investigate the association between primary Sjögren’s syndrome (pSS) and coronary heart disease (CHD), and the influence of medications for pSS patients on risk of CHD. The authors identified 4175 patients with a new diagnosis of pSS between 2002 and 2013 from the National Health Insurance Research database. The control-to-case ratio was 4:1. The risk and cumulative incidences of CHD were calculated. The adjusted hazard ratio (HR) of CHD for pSS patients was 1.17 (1.03–1.34) after adjusting for age, sex, comorbidities, and medications. The cumulative incidence for CHD in the pSS group was significantly higher than that in the control group (log-rank p < 0.0001). The risk of CHD in pSS patients was increased with age by 4% per year, and 45- to 59-year-olds were at the highest risk (HR = 1.464, 1.195–1.794). The application of corticosteroids (HR = 1.45, 1.07–1.97) as well as NSAIDs (HR = 1.31, 1.05–1.65) both increased the risk of CHD among pSS patients. pSS is associated with an increased risk of subsequent CHD in Taiwan. Primary Sjögren’s syndrome might be an independent risk factor for CHD. Use of corticosteroids and NSAIDs in the treatment of pSS patients increased the risk of developing CHD.

## Introduction

Primary Sjögren’s syndrome (pSS) is a chronic systemic autoimmune disease characterised by xerostomia and xerophthalmia caused by focal lymphocytic infiltration in the salivary and lacrimal glands^1^. The prevalence of pSS is estimated to be between 0.05% and 4.8% of the world population, and the female-to-male ratio is 9:1^2^. The adjusted standardised mortality ratio in patients with pSS is 1.38, with no significant increase in all-cause mortality compared with the general population^3^. As for the causes of mortality, cardiovascular disease (CVD), solid-organ and lymphoid malignancies and infections have been reported to be the leading causes of death in pSS patients^3,4^. It is known that, compared to the general population, patients with pSS have an increased risk of overall malignancy, especially non-Hodgkin’s lymphoma, and this risk may be up to 13.78-fold higher^5^. However, it is not yet clear whether pSS confers an increased risk of CVD or whether these were just representative of usual causes of death in an ageing population. Meanwhile, although pSS can affect lung, skin, kidney and almost every organ, little research has reported cardiovascular involvement.

Among all the cardiovascular diseases, coronary heart disease (CHD) secondary to coronary atherosclerosis is the leading cause of mortality worldwide. In 2012 CHD and stroke respectively accounted for 42.3% and 38.3% of all cardiac deaths, and both of these originate from atherosclerosis^6^. Many risk factors are involved in the development of atherosclerosis, such as hypertension, hyperlipidaemia, diabetes, smoking, etc. However, these traditional risk factors do not fully explain the whole of coronary heart disease, for many studies have identified the role of inflammation^7,8^. In recent years, there has emerged substantial evidence linking systemic autoimmune disorders such as systemic lupus erythematosus (SLE) and rheumatoid arthritis (RA) with increased risk of coronary atherosclerosis^9–11^. Furthermore, intriguing observations of RA patients showing a non-linear relationship of traditional cardiovascular risk factors with both atherosclerosis progression and cardiovascular mortality in the general population suggest that multiple factors may play a role in this process. Autoimmune disease itself is likely to be one independent risk factor for atherosclerosis due to underlying chronic inflammation^12^.

pSS is similar to SLE and RA in terms of several clinical, inflammatory and immunological characteristics. However, the association between pSS and CHD remains controversial. Previous studies have demonstrated that pSS patients have increased cardiovascular disease risk factors such as hypertension, hyperlipidaemia, diabetes, etc^12–15^. Nevertheless, inconsistent findings regarding subclinical atherosclerosis and endothelial dysfunction measured by different methods in pSS patients have also been described^16–18^. Further, population-based surveys from different countries investigating the association between pSS and CHD risk were inconsistent^9,12,19^. Disease duration seems to play a role in this discrepancy^20,21^, as Rachapalli *et al*. demonstrated that ankle-brachial index, a simple method of identifying subclinical atherosclerosis, was not significantly reduced in pSS patients except for those patients with a disease duration of more than 10 years^21^. Thus, a large-scale study with a long follow-up duration is needed to validate the risk of CHD in patients with Sjögren’s syndrome.

In the present study, we conducted a 12-year nationwide population-based retrospective cohort analysis to investigate the association between pSS and CHD. Moreover, we also investigated the influence of medications, such as corticosteroids, disease-modifying antirheumatic drugs (DMARDs), nonsteroidal antiinflammatory drugs (NSAIDs), and other medicines used in pSS patients at risk of CHD.

## Results

### Characteristics of the study subjects

The characteristics of the enrolled 4175 patients with pSS and 16,700 controls after age and gender matching are depicted in Table 1. The mean age at the time of diagnosis of pSS was 50.15 ± 16.80 years, and the female to male ratio was nearly 3:1 (75.40% vs. 24.60%). Compared with the controls, patients with pSS were more likely to have all the comorbidities we had considered including DM (12.19% vs. 9.19%), hypertension (22.87% vs. 19.68%), hyperlipidaemia (17.56% vs. 12.71%), COPD (17.87% vs. 11.07%), and stroke (4.13% vs.2.82%), etc. Consistent with the above results, most medications for comorbidities were more frequently used in the pSS group than the control group, except for the antihyperglycemic drugs.

**Table 1:** Baseline characteristic among patients with primary Sjögren’s syndrome and controls.

	Controls n = 16,700	Sjögren’s syndrome n = 4,175	p-value
Age at baseline	50.12 ± 16.80	50.12 ± 16.80	1.0000
Sex			1.0000
Female	12,592(75.40%)	3,148(75.40%)	
Male	4,108(24.60%)	1,027(24.60%)	
Co-morbidities			
Diabetes mellitus	1535(9.19%)	509(12.19%)	**<0.0001**
Hypertension	3286(19.68%)	955(22.87%)	**<0.0001**
Hyperlipidaemia	2122(12.71%)	733(17.56%)	**<0.0001**
COPD	1848(11.07%)	746(17.87%)	**<0.0001**
Stroke	480(2.87%)	179(4.29%)	**<0.0001**
Chronic kidney disease	406(2.43%)	133(3.19%)	**0.0060**
Alcohol-Related Disease	87(0.52%)	34(0.81%)	**0.0255**
Chronic liver diseases	1118(6.69%)	540(12.93%)	**<0.0001**
Cancer	591(3.54%)	210(5.03%)	**<0.0001**
Medications			
Antihyperglycemic drugs	1040(6.23%)	287(6.87%)	0.1256
Antihypertensive drugs	2852(17.08%)	952(22.80%)	**<0.0001**
Statin	691(4.14%)	203(4.86%)	**0.0386**
Corticosteroids (oral)	371(2.22%)	464(11.11%)	**<0.0001**
NSAID	1928(11.54%)	1031(24.69%)	**<0.0001**
DMARDs*	9(0.05%)	657(15.74%)	**<0.0001**
Aspirin	541(3.24%)	203(4.86%)	**<0.0001**
PPI	212(1.27%)	160(3.83%)	**<0.0001**
H2 Receptor	485(2.9%)	262(6.28%)	**<0.0001**

COPD, chronic obstructive pulmonary disease; DMARDs, disease-modifying antirheumatic drugs; NSAID, nonsteroidal antiinflammatory drugs; PPI, proton pump inhibitors.

*DMARDs, including Hydroxychloroquine, Methotrexate, Sulfasalazine.

### Incidence of CHD among pSS patients and controls

**Figure 1:**
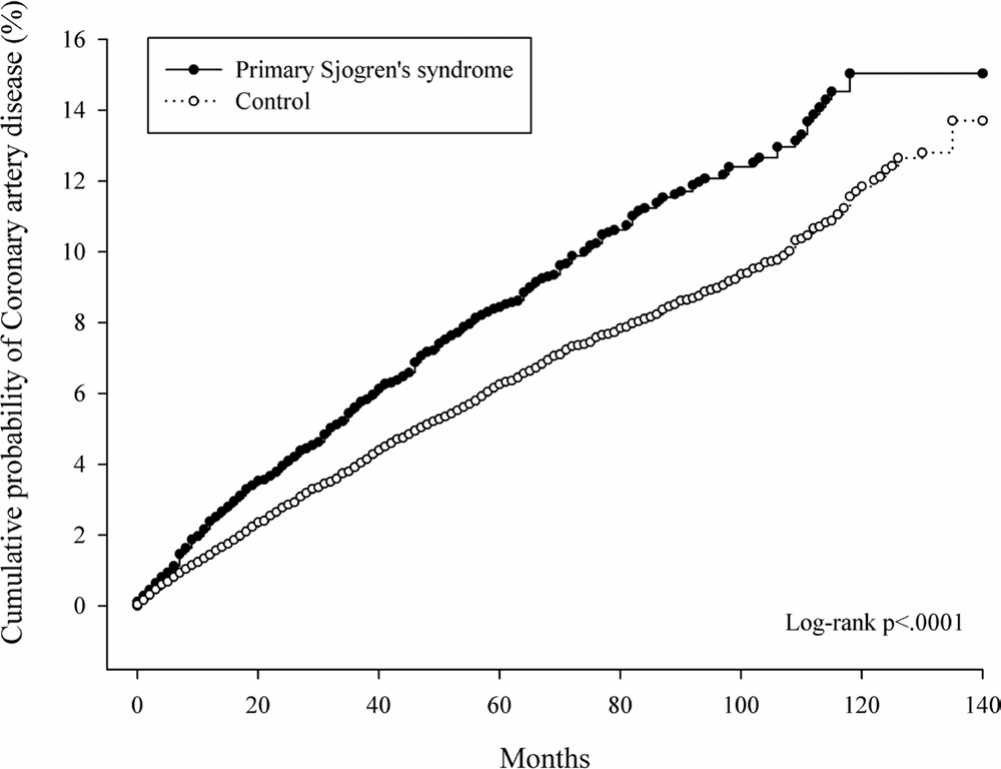
Cumulative probability of coronary heart disease between pSS patients and controls. After a maximum 140-month follow-up period, the cumulative incidence of CHD in the pSS group was significantly higher than that in the control group (p < 0.0001).

A total of 365 pSS patients and 1090 controls were identified as having CHD, corresponding to 1.42 and 1.04 per 1000 person-months during the observation period totalling 256,883 and 1,043,101 person-months for the pSS and control groups, respectively (Table 2). The incidence rate of CHD in the pSS group was 1.36 (95% CI = 1.21–1.53) times higher than that in the control group (p < 0.0001, Table 2). After approximately 12 years follow-up, the cumulative incidence for CHD in the pSS group was significantly higher than that in the control group (log-rank p < 0.0001, Fig. 1).

**Table 2:** Incidence rate of coronary heart disease in primary Sjögren’s syndrome and control group.

	Controls n = 16,700	Sjögren’s syndrome n = 4,175	p value
Follow up person-months (min, max, median)	1,043,101 (1,140,60)	256,883 (1,140,59)	
Event of CAD	1,090	365	
Incidence rate (per 1000 person-months)	1.04	1.42	
Incidence rate ratio (95% C.I.)	Reference	1.36(1.21–1.53)	**<0.0001**

CHD, coronary heart disease; C.I, confidence interval.

### Risk for CHD among pSS patients and controls

Compared with the control group, pSS patients had 1.17 or 1.24-fold risk after adjusting for age, sex, comorbidities, and medications or not (95% CI = 1.03–1.34 and 1.10–1.40, respectively) (Table 3). The risk of CHD increased with age, and the risk increased 1.04-fold (95% CI = 1.04–1.05) for each additional year (Table 3). Men had 1.14 times the risk of women (Table 3). Patients with DM, hypertension, hyperlipidaemia, and COPD were at increased risk of CHD, with adjusted HRs of 1.16 (95% CI = 1.01–1.34), 1.70 (95% CI = 1.51–1.92), 1.38 (95% CI = 1.21–1.57) and 1.32 ((95% CI = 1.16–1.50), respectively (Table 3). After excluding those patients with comorbidities associated with CHD, the adjusted HR of pSS for CHD changed to 1.52 (95% CI = 1.21–1.92, Table 4). Considering the impact of drugs on the risk of CHD, we found that people using NSAIDs (HR = 1.26, 95% CI = 1.11–1.43) and aspirin (HR = 1.45, 95% CI = 1.20–1.74) had a higher risk of CHD (Table 3), whereas other drugs including corticosteroids, seemed to have nothing to do with the occurrence of CHD (Table 3).

**Table 3:** The risk for CHD between patients with pSS and control group in Cox proportional hazard regression modelling.

	Model 1^*^			Model 2^#^		
	aHR*	95% C.I.	p value	aHR*	95% C.I.	p value
Sjögren’s syndrome	1.24	1.10–1.40	**0.0005**	1.17	1.03–1.34	**0.0140**
Age (per 1 year)	1.04	1.04–1.04	**<0.0001**	1.04	1.04–1.04	**<0.0001**
Sex (reference: Female)						
Male	1.14	1.01–1.28	**0.0327**	1.15	1.02–1.29	**0.0218**
Co-morbidities						
Diabetes mellitus	1.16	1.01–1.34	0.0371	1.15	0.93–1.41	0.1956
Hypertension	1.70	1.51–1.92	<0.0001	1.55	1.32–1.82	<0.0001
Hyperlipidaemia	1.38	1.21–1.57	<0.0001	1.39	1.21–1.59	<0.0001
COPD	1.32	1.16–1.50	<0.0001	1.27	1.11–1.44	0.0003
Stroke	1.07	0.86–1.31	0.5522	0.98	0.79–1.22	0.8705
Chronic kidney disease	1.17	0.93–1.48	0.1818	1.17	0.92–1.48	0.1952
Chronic liver diseases	1.21	1.03–1.42	0.0175	1.19	1.02–1.4	0.0294
Cancer	0.98	0.77–1.25	0.8759	0.98	0.77–1.24	0.8606
Medications						
Antihyperglycemic drugs				0.99	0.78–1.26	0.9428
Antihypertensive drugs				1.07	0.91–1.26	0.3934
Statin				0.86	0.69–1.06	0.1604
Corticosteroids (oral)				1.20	0.97–1.49	0.0963
NSAID				1.26	1.11–1.43	**0.0004**
DMARDs^$^				1.00	0.73–1.35	0.9786
Aspirin				1.45	1.20–1.74	**<**.0001
PPI				0.96	0.68–1.35	0.7963
H2 Receptor				1.15	0.92–1.43	0.2191

aHR, adjusted hazard ratio; C.I, confidence interval; COPD, chronic obstructive pulmonary disease; DMARDs, disease-modifying antirheumatic drugs; NSAID, nonsteroidal antiinflammatory drugs; PPI, proton pump inhibitors.

*In model 1, aHRs were adjusted for age, sex, and co-morbidities. ^#^In model 2, aHRs were adjusted for age, sex, co-morbidities, and medications.

^$^DMARDs, including Hydroxychloroquine, Methotrexate, Sulfasalazine.

**Table 4:** Hazard ratios of CAD with potential risks in specific sub-groups.

	Subgroup 1: Excluded the individuals with high risk of CVD			Subgroup 2: Only involved the SS patients		
	aHR*	95% C.I.	p value	aHR*	95% C.I.	p value
Sjögren’s syndrome	1.52	1.21–1.92	**0.0004**	—	—	—
Age (per 1 year)	1.06	1.05–1.06	**<0.0001**	1.04	1.03–1.04	**<0**.0001
Sex (reference: Female)						
Male	1.01	0.82–1.24	0.9364	1.06	0.84–1.35	0.6137
Co-morbidities						
Diabetes mellitus	—	—	—	0.90	0.60–1.34	0.5898
Hypertension	—	—	—	1.72	1.28–2.32	0.0003
Hyperlipidaemia	—	—	—	1.15	0.87–1.51	0.3340
COPD	—	—	—	1.22	0.96–1.55	0.0985
Stroke	0.70	0.29–1.70	0.4277	1.05	0.70–1.58	0.8221
Chronic kidney disease	1.72	0.91–3.23	0.0944	0.99	0.58–1.67	0.9548
Chronic liver diseases	1.08	0.73–1.59	0.7038	1.08	0.81–1.44	0.5865
Cancer	0.86	0.51–1.47	0.5883	1.11	0.72–1.72	0.6417
Medications						
Antihyperglycemic drug	—	—	—	1.13	0.69–1.84	0.6393
Antihypertension drug	—	—	—	0.87	0.65–1.16	0.3415
Statin	—	—	—	1.28	0.84–1.95	0.2440
Corticosteroids (oral)	1.59	0.99–2.58	0.0569	1.45	1.07–1.97	**0.0175**
NSAID	1.46	1.12–1.89	0.0045	1.31	1.05–1.65	**0.0192**
DMARDs^$^	0.66	0.34–1.26	0.2051	0.92	0.66–1.27	0.5979
Aspirin	—	—	—	0.99	0.67–1.46	0.9626
PPI	1.64	0.80–3.37	0.1774	0.83	0.48–1.43	0.5060
H2 Receptor	1.32	0.82–2.14	0.2520	1.17	0.80–1.71	0.4084

aHR, adjusted hazard ratio; C.I, confidence interval; COPD, chronic obstructive pulmonary disease; DMARDs, disease-modifying antirheumatic drugs; NSAID, nonsteroidal antiinflammatory drugs; PPI, proton pump inhibitors.

*aHR, adjusted hazard ratio, adjusted for age, sex, co-morbidities, and medications.

^$^DMARDs, including HCQ, Methotrexate, Sulfasalazine.

Subgroup 1 excluded the patients had any of DM, hypertension, hyperlipidaemia, COPD or use of anti-hyperglycaemic drugs, antihypertensive drugs, statin and aspirin from all participations. The 2,010 patients with SS and 5,788 controls were excluded from analysis.

Subgroup 2 The analysis was performed only in SS group.

### Risk of CHD in specific sub-groups of pSS patients

**Figure 2:**
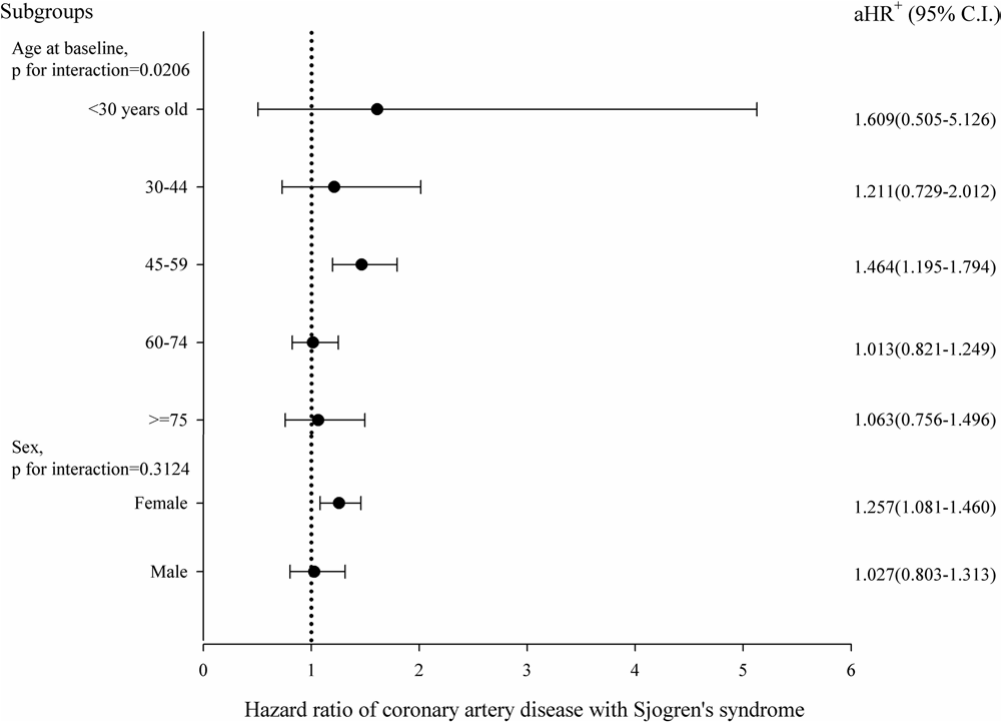
Hazard ratios of coronary artery disease with exposure of Sjogren’s syndrome stratified by age groups or sex. The risk of CHD in pSS patients aged 45–59 years old had the highest risk of CHD (HR = 1.46, 95% CI = 1.20–1.79), the interaction p = 0.02. The risk for female pSS patients was higher than that for male patients (HR = 1.26, 95% CI = 1.08–1.46) (Fig. 2), but there was no significant interaction (interaction p = 0.31) between sex and pSS exposure on CHD. aHR+, adjusted hazard ratio, was controlled by age, sex, co-morbidities, and medications.

To determine which pSS patients were most susceptible to CHD, stratified analyses were conducted in the pSS group. The risk of CHD in pSS patients increased with age, by 6% per year (Table 4), and patients aged 45–59 years old had the highest risk of CHD (HR = 1.46, 95% CI = 1.20–1.79), with an interaction p = 0.02 (Fig. 2). The risk for female pSS patients was higher than that of male patients (HR = 1.26, 95% CI = 1.08–1.46), but there was no significant interaction (interaction p = 0.31) between sex and pSS exposure on CHD (Fig. 2). The risk of developing CHD for pSS patients with comorbidities was not higher than that for pSS patients without corresponding comorbidities (Table 4, Supplement Fig. 1), whereas the application of corticosteroids (HR = 1.45, 95% CI = 1.07–1.97), and NSAIDs (HR = 1.31, 95% CI = 1.05–1.65) did increase the risk of CHD among pSS patients (Table 4).

## Discussion

The present study revealed an increased risk of CHD among pSS patients compared with age- and sex-matched controls. In addition, after excluding all the patients complicated with DM, hypertension, hyperlipidaemia, COPD and those who used anti-hyperglycaemic, antihypertensive drugs, statin and aspirin, the adjusted HR of pSS for CHD increased to 1.52, suggesting that primary Sjögren’s syndrome may itself be an independent risk factor for CHD, as is hypertension.

Primary Sjögren’s syndrome is one of the most prevalent autoimmune diseases predominantly affecting the lacrimal and salivary glands, resulting in sicca symptoms. In addition to affecting the exocrine glands, systemic involvement can also frequently occur, usually representing a relatively poor prognosis. pSS is a chronic, slow-evolving, and a non-life-threatening illness in the vast majority of patients, with 5-and 10-year cumulative survival rates following diagnosis of 95% and 90%, respectively^22^. Cardiovascular diseases has been confirmed as the leading cause of death, which is in contrast to the previous thinking that lymphoma was the leading cause of mortality in these patients^3^. Among all of the cardiovascular diseases, coronary heart disease secondary to coronary atherosclerosis is the leading cause of mortality worldwide.

To our knowledge, several studies from different countries have assessed the association between pSS and cardiovascular disease risk (summarised in Table 5), but no consistent conclusion has been drawn^9,12,14,19,23,24^. The endpoints on which these studies focused were either coronary arterial disease or stroke (ischaemic or haemorrhagic). Both groups from Taiwan and Sweden conducted corresponding research on both endpoints but acquired different results. The risk found for coronary heart disease, ischaemic and haemorrhagic stroke was increased in pSS patients compared with the general population among patients from Sweden hospitalised for immune-mediated disease between 1987 and 2008^9,24^. Data from a previous Taiwanese cohort collected between 2002 and 2006, however, did not confirm higher risk of acute myocardial infarction and ischaemic stroke among pSS patients^19,23^. Another population-based study from Italy compared the prevalence of myocardial infarction, cerebrovascular events and heart failure between a subgroup of 788 female patients with pSS and 4774 age-matched healthy women and found that cerebrovascular events and myocardial infarction were more common in patients with pSS^12^. A single-centre case-control Spanish study showed similar contrasts in prevalence rates of ischaemic heart disease and stoke between pSS patients and controls^14^. In our research, we chose CHD as the endpoint to investigate the association between pSS and mortality from cardiovascular diseases. Our results were similar to the study in Sweden that the risk for CHD was higher among pSS patients compared with the general population, but they were different from the previous Taiwanese study. First, the databases that we used were different. We chose the LHID 2000, whereas they used the Catastrophic Illness Patient (RCIP) database. The RCIP database contains health claim data for people with 31 medical conditions including pSS. Patients with pSS in the RCIP database had their medical, laboratory, and/or pathological reports reviewed according to the 2002 American-European Consensus Group classification criteria for a catastrophic illness certificate. As such, the RCIP database is considered very accurate for the diagnosis of pSS. However, it was more likely to have been influenced by the health insurance system. For example, the number of patients included in one year might have been more susceptible to one particular drug and thus might not truly have reflected the true conditions. This prompted us to switch to the LHID 2000 database, which is also very accurate for diagnosing autoimmune diseases and cardiovascular diseases. Second, the endpoints were not exactly the same. They only investigated the occurrence of acute myocardial infarction (AMI), whereas we observed all coronary heart diseases, including AMI. In addition, the follow-up period for our study was much longer (maximum follow-up duration, 12 years vs. 5 years). Based on these differences, some noteworthy points should be addressed. First, the association seems to be profoundly influenced by follow-up duration. If the mean duration of follow-up is not long enough (<5 years), pSS may not show an association with risk of CVD. Second, the fact that some studies may have mixed populations of primary and secondary SS could have largely influenced the final results. Third, the significant association between pSS and risk of CVD must be confirmed in both population-based and hospital-based studies.

**Table 5:** Studies investigating the cardiovascular diseases risk in primary Sjögren’s syndrome.

Study	Region	Study design and setting	Study period; mean follow-up years(years)	Participants (n)	Diagnostic criteria	Mean age(years)	Sex, n	Cardiovascular diseases	Results
Perez-De-Lis, M. *et al*.^14^	Spain	case-control; single-centre	1984–2009; NA	pSS(312); HC(312)	2002 AECG criteria	NA	NA	ischemic heart disease; stroke	The prevalence of cardiovascular disease was similar in pSS patients and controls.
Zoller, B. *et al*.^24^	Sweden	retrospective cohort; population-based	1987–2008; NA	SS(1300); controls(NA)	ICD	NA	M 125; F 1175	ischemic and haemorrhagic stroke	Hospitalization for SS is associated with increased risk of haemorrhagic stroke.
Zoller, B. *et al*.^9^	Sweden	retrospective cohort; population-based	1964–2008; NA	SS(1,420); total population of Sweden controls	ICD	NA	M 136; F 1284	CHD	pSS are associated with increased risk of CHD after hospital admission.
Chiang, C.H. *et al*.^19^	Taiwan	retrospective cohort; population-based;	2002–2006; 3.7 ± 1.8	pSS(5,205); general population controls(5,205)	ICD	53.2 ± 14.1	M 651; F 4554	AMI	pSS is not associated with a higher risk of subsequent AMI.
Chiang, C. H. *et al*.^23^	Taiwan	retrospective cohort; population-based;	2002–2006; 3.7 ± 1.9	pSS(4,276); general population controls(42,760)	ICD	51.2 ± 13.7	M 496; F 3750	ischemic stroke	pSS is not associated with a higher risk of subsequent ischemic stroke.
Bartoloni, E. *et al*.^12^	Italy	retrospective cohort; population-based	NA; 5 ± 6	pSS(1,343); healthy women controls(4,774)	1993 European Community Study Group diagnostic	57 ± 14	M 59; F 1284	MI, cerebrovascular events and heart failure.	pSS is associated with an increased risk of cerebrovascular events and MI. In the whole population, central nervous system involvement and use of immunosuppressive therapy were associated with a higher risk of cardiovascular events.

AECG, American-European Consensus Group; AMI, acute myocardial infarction; CHD, coronary heart disease; F, female; HC, healthy control; ICD, International Classification of Diseases; M, male; MI, myocardial infarction; NA, not available; pSS, Primary Sjögren’s syndrome.

It is well-known that coronary atherosclerosis is the underlying condition for acute coronary events with few exceptions. In pSS, higher subclinical atherosclerosis or endothelial dysfunction has been described using different methods and different parameters (summarised in Supplementary Table S1)^16–18,20,21,25–32^. An intima-media thickness (IMT) score >0.90 mm in the carotid and femoral arteries was the most frequently used indicator to reflect the presence of subclinical atherosclerosis. Vaudo *et al*.^16^ were the first to observe the high IMT scores in pSS patients, and this result has been reproduced by two other groups^16,17,25^. However, there are three studies that have shown similar IMT values among pSS patients and controls^27,30,31^. These different results appear to be partly influenced by the different diagnostic criteria used to identify pSS. Patients with pSS diagnosed by 2002 AECG criteria and 2012 ACR criteria did not show a significant association. In addition, disease duration was also a possible important reason for the differences. The disease duration for the three studies that found increased IMT scores associated with pSS was much longer than those that did not. Other parameters used to detect subclinical atherosclerosis included ankle-brachial index (ABI), aortic stiffness, endothelial-dependent flow-mediated dilation (FMD) and endothelial-independent nitrate-mediated vasodilatation (NMV). Although the results may be a bit inconsistent, taken all together, it appears that pSS conveys a higher risk for impaired endothelial function and subclinical atherosclerosis. Admittedly, the sample size of these studies is too small to draw firm conclusions, and the association should be further investigated in a larger number of patients with pSS using various diagnostic criteria.

Despite the increased risk of subclinical atherosclerosis in pSS patients, coronary atherosclerosis is in fact a common condition in the general population. Autopsy series in US communities of young adults (mean age, 36 ± 14 years) who died of non-natural causes revealed coronary atherosclerosis in >80% of the autopsy sample, with ≈8% having obstructive disease. Thus most people ≥40 years of age in our society have coronary atherosclerosis^33,34^. However, the annual incidence of acute coronary events is rather low given the high prevalence of coronary atherosclerosis^35^. The reason for this is that the occurrence of acute coronary events requires a thrombosis-promoting milieu, resulting a clinically significant reduction on coronary blood flow and associated myocardial ischaemia in addition to rupturing of coronary atherosclerotic plaques^33,35^. However, there is also compelling evidence that plaque rupture and thrombus formation most often do not lead to acute coronary events^33,36^. There usually exists three possible conditions following plaque rupture. In the vast majority of cases, plaque rupture leads to plaque healing and growth. In some cases, thrombus material is embolised distally, which may cause symptoms of coronary arterial insufficiency or asymptomatic microinfarctions. Only if plaque rupture coincides with a thrombosis-conducive state at the site of plaque rupture or erosion may arterial thrombosis and occlusion occur, which may then trigger a coronary event^33,37,38^. A study by Bartoloni E. *et al*. detected increased circulating endothelial microparticles and endothelial progenitor cells in pSS, demonstrating that existing endothelial dysfunction as well as that plaque rupture in pSS patients tends not to cause thrombosis or acute coronary events^39^. Therefore, when exploring the mortality of cardiovascular diseases in pSS, the risk of acute coronary events, such as CHD, might be a better observation index than the incidence of atherosclerosis.

Our findings, in line with some previous studies, confirm an increased risk of CHD in pSS patients, but we all know that multiple factors are associated with the incidence of CHD, including hypertension, hyperlipidaemia, diabetes, smoking, as well as modifiable lifestyle habits such as unhealthy diet^40^. Moreover, previous articles have confirmed that, despite differences in the selection of controls and regions of study, pSS is indeed associated with these cardiovascular risk factors (summarised in Supplementary Table S2)^12–15,41–44^. Our results also show that traditional cardiovascular risk factors such as DM, hypertension, hyperlipidaemia, smoking, and alcohol, are more common in pSS patients than in the general population. All these findings suggest that either the disease itself or the increased risk factors represent a risk for developing CHD. We thus excluded all patients with DM, hypertension, hyperlipidaemia, and COPD or those who used anti-hyperglycaemic drugs, antihypertensive drugs, statin or aspirin to avoid interference by these traditional cardiovascular risk factors. We found that the adjusted HR of pSS for CHD increased to 1.52 (1.21–1.92), confirming that primary Sjögren’s syndrome may itself be an independent risk factor for CHD, just as is hypertension. Many factors may play a role in this association. For example, autoantibodies anti-Ro-SSA or anti-La-SSB, which are markers of Sjögren’s syndrome, have been shown to be associated with higher IMT scores and lower ABI and NMV^16,18,29^. The patient subset with leukopenia (a haematological marker associated with more severe disease) had been demonstrated a sixfold higher risk of developing angina compared to those with a normal leukocyte count^12^. Unfortunately, due to the limitations of the database that we used, we could not further validate the role of specific disease characteristics in our research, which is one of our limitations. Therapies for autoimmune disorders, particularly corticosteroids, which are associated with metabolic syndrome and premature atherosclerosis, have always been considered an important factor contributing to increased risk of cardiovascular diseases in rheumatic diseases. In our study, we explored the relationship between drugs commonly used in the treatment of pSS and risk of CHD, finding that the application of corticosteroids (HR = 1.45, 1.07–1.97) indeed increased the risk. Our research is the first population-based study to demonstrate the effect of corticosteroid on risk of CHD in pSS patients. In addition, we found that NSAIDs (HR = 1.31, 1.05–1.65) also increased the risk. These results underline the need for routine assessment of CHD risk during follow-up of patients with pSS.

Until now, the possible mechanism of CHD in pSS as well as other rheumatic diseases has remained unknown. A current unifying view of atherosclerosis pathophysiology is that CHD is a chronic inflammatory disease^45^. In the artery wall, the retention of plasma lipoproteins in endothelium triggers monocyte-derived macrophages and T helper type 1 (Th1) cells to form atherosclerotic plaques. Inflammation is initiated by innate immune response of modified lipoproteins and is perpetuated by Th1 cells that react with autoantigens from the apolipoprotein B100 protein of LDL. Other T cells are also active in atherosclerotic lesions. Regulatory T cells inhibit pathological inflammation, whereas Th17 cells promote plaque fibrosis^45^. All of the cells involved in the atherosclerotic process secrete and are activated by cytokines. Thus, inflammation plays a key role in and may transduce the effects of many known risk factors for the disease^46^. An increasing number of articles have clearly confirmed the increased risk of CHD in patients with RA and SLE^7,9–11^. Primary Sjögren’s syndrome is also a systemic inflammation with peripheral changes in cytokines and immune cells. Perhaps this systemic change may affect plaque lesions. The specific mechanism of increased risk of CHD in pSS patients needs to be further explored.

Several limitations should be noted in this study. First, there were no laboratory data available in the claims data to which our study referred. We thus could not analyse in detail factors predicting CHD. Second, the median disease duration that was data-tracked (5 years) in this study neglects the period prior to diagnosis. Third, we included patients with pSS from the LHID 2000 database. Thus, some patients who may not have been adequately diagnosed with pSS were included in the cohort. Fourth, that pSS patients were in regular contact with the health care system make them more likely or easier to be diagnosed with CHD than those from the general population who were less likely to interact with the health care system. However, the strengths of this study are also obvious. Our findings were based on the analysis of a large sample from a nationwide population-based dataset, which minimised selection bias and was representative of the general population. The follow-up duration of this study was 12 years, long enough to observe subsequent CHD occurrence. Moreover, we further validated the association between pSS and CHD on the basis of limiting interference from traditional risk factors. In addition, our study was the first to investigate the relationship between drugs commonly used in the treatment of pSS and the risk of CHD. Overall, our research was a well-designed and convincing investigation. In consideration of the above-mentioned limitations, the risk of CHD was underestimated in this study.

In conclusion, our results showed that pSS is associated with an increased risk of subsequent CHD in Taiwan. Primary Sjögren’s syndrome might itself be an independent risk factor for CHD. Patients with pSS aged 45–59 years were at the highest risk of CHD compared with their counterparts. Use of corticosteroids and NSAIDs for the treatment of pSS patients increased the risk of developing CHD. Further studies are needed to investigate possible mechanisms associated with pSS and CHD.

## Methods

### Data sources

The data used in this study came from the 2000 Longitudinal Health Insurance Database (LHID 2000), a subset of the National Health Insurance Research database (NHIRD) in Taiwan. The NHIRD database consists of all inpatient and outpatient visits, procedure codes, catastrophic illness files, and drug prescription data of the 23.5 million insured residents, whereas the LHID 2000 contains all the original claims data for one million beneficiaries randomly sampled from the 2000 Registry for Beneficiaries of the National Health Insurance programme. The 2000–2013 records of outpatient visits, admission, and prescriptions were retrieved for analysis. There are no significant differences in age, gender and health care costs between the LHID and NHIRD^47^.

The diseases discussed in this paper were defined in the NHIRD based on the International Classification of Diseases, Ninth Revision (ICD-9) code, which has been shown to be highly sensitive and specific for the diagnosis of autoimmune rheumatic diseases including pSS^48^. Moreover, the NHIRD is a valid resource for population research on cardiovascular diseases, as the validity of acute myocardial infarction (AMI) diagnosis coding in the NHIRD has been demonstrated^49^. Therefore the diagnosis of patients included in this study was highly accurate and reliable. This study was approved by the Ethics Review Board of Chung Shan Medical University (CS15134). Patient informed consent was not required, as the NHIRD data files contain only de-identified secondary data.

### Study subjects

We identified new cases of Sjögren’s syndrome (ICD-9 code: 710.2) who had at least 3 outpatients diagnosis within 2 years or 1 admission diagnosis between 2002 and 2013. The first diagnosis date was defined as the index date. Patients with autoimmunity disease history [including RA (ICD-9 code: 714.0), SLE (ICD-9 code: 710.0), psoriasis (ICD-9 code: 696.0, 696.1), ankylosing spondylitis (ICD-9 code: 720.0) and some other diffuse diseases of the connective tissue (ICD-9 code: 710.1, 710.3, 710.4)], who should be classified as secondary SS (sSS) were excluded from the study. We also excluded patients who died within 90 days after the index date. Our purpose in this study was to investigate the causal relationship between pSS and CHD; thus patients who had CHD before or within 90 days after the index date were excluded. CHD was defined in this study as ≥2outpatient diagnoses or ≥1admission diagnosis of CHD (ICD-9 code: 410–414).

Controls were randomly selected from patients without SS (pSS or sSS) and at risk for CHD. They were individually-matched with pSS patients at a 4:1 ratio based on sex and age. The index date for each control was assigned based on matched pSS cases. The comorbidities considered in the study included hypertension (ICD-9 code: 401–405), diabetes mellitus (DM) (ICD-9 code: 250), hyperlipidaemia (ICD-9 code: 272), chronic obstructive pulmonary disease (COPD) (ICD-9 code: 490–496), alcohol-related disease (ICD-9 code: 291, 303, 305.0, 357.5, 425.5, 535.3, 571.0–571.3, 790.3, 977.3, 980.0), stroke (ICD-9 code :430–436), chronic kidney disease (ICD-9 code: 582, 583, 585, 586, 588), chronic liver diseases (ICD-9 code: 456.0–456.2, 571.2, 571.4–571.6, 572), and cancer(ICD-9 code: 140–208). All comorbidities were identified when diagnosed within 2 years prior to the index date. Medications for treating hypertension, diabetes mellitus, and hyperlipidaemia, as well as drugs for the treatment of pSS, including corticosteroids, DMARDs, NSAIDs, and symptom-modifying drugs taken for at least 14 days within 90 days of the index date were all taken into account of in this study. All study subjects were followed from 90 days following the index date until CHD occurrence, death or the end of 2013 whichever came first.

### Statistical analysis

Differences between case and control groups in comorbidities and medications distribution were compared with Chi-square tests. To assess the risk of CHD during the follow-up period, Cox proportional hazard regression model was used to estimate Hazard ratio (HR) and 95% confidence intervals (95% CI). The cumulative incidence for CHD in both groups was also plotted using Kaplan-Meier analysis, and differences were tested using log-rank testing. A two-tailed p value of <0.05 was considered statistically significant. All statistical analyses were conducted using SAS Statistics software (version 9.4; SAS Institute, Inc., Cary, NC, USA).

### Availability of materials and data

All data generated or analysed during this study are included in this article (and its Supplementary Information files).

## Electronic supplementary material

Supplementary files
