# Supplementary material for: Increased risk of coronary heart disease among patients with primary Sjögren’s syndrome: a nationwide population-based cohort study
Source: Sci Rep. 2018 Feb 2;8:2209. doi: 10.1038/s41598-018-19580-y (PMC5797247; doi:10.1038/s41598-018-19580-y)
Supplement: Supplementary file 1 — Supplementary files [file 41598_2018_19580_MOESM1_ESM.doc]

**Increased** **risk of coronary heart disease among patients with primary** **Sjögren's** **syndrome: a** **nationwide population-based cohort study**

**Xue-Fen Wu^1^, Jing-Yang Huang^2^, Jeng-Yuan Chiou^3^, Huang-Hsi Chen^4^，James Cheng-Chung Wei^5^*, Ling-Li Dong^1^***

**Figure S1** Hazard ratios of coronary artery disease with exposure of Sjogren`s syndrome stratified by co-morbidities.


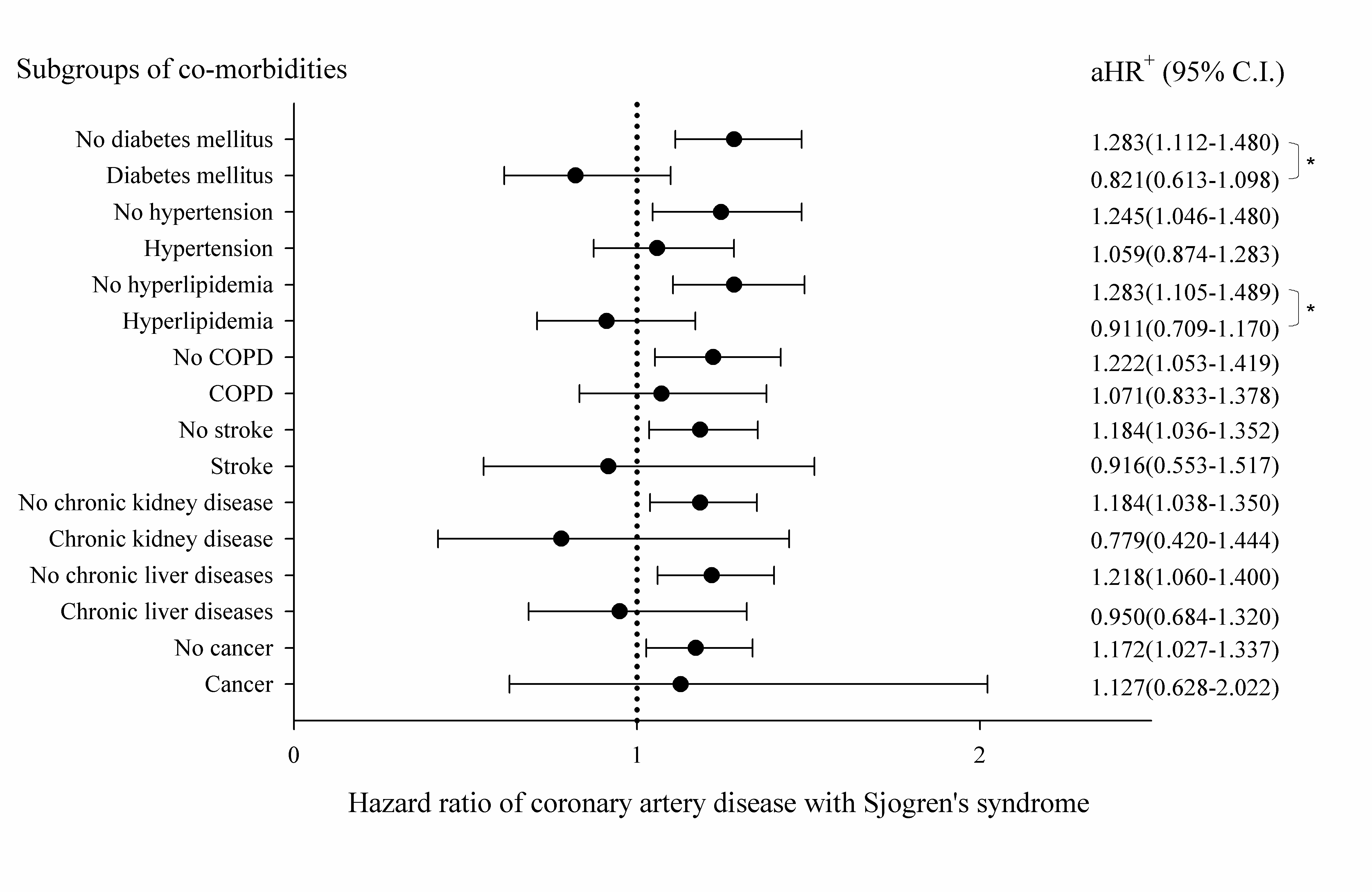


Comorbidities complicated with pSS patients would not increase the risk of developing CHD compared with pSS patients without corresponding comorbidities

aHR^+^, adjusted hazard ratio, was controlled by age, sex, other co-morbidities, and medications.

^*^ The test for interaction was significance.

**Table S1** Studies investigating endothelial dysfunction, subclinical atherosclerosis in primary Sjögren’s syndrome.

| study | region | study design and setting | disease duration (years) | participants（n） | diagnostic criteria | mean age (years) | sex, n | Subclinical Atherosclerosis | results |
| --- | --- | --- | --- | --- | --- | --- | --- | --- | --- |
| Vaudo, G. et al, 2005^1^ | Italy | case-control; single-center | 10.9（median） | pSS(37); HC(35) | 2002 AECG criteria | 48±14 | M 0;  F 37 | IMT | Subclinical ATS was evident in about one-half of the patients with SS. Anti-SSA antibodies were independent predictors of carotid artery thickening, while leukopenia was a predictor of both carotid and femoral artery thickening |
| Pirildar T. et al, 2005^2^ | Turkey | case-control; single-center | 2.92(median) | pSS(25); HC(29) | 1996 European classification criteria | 47.1±9.7 | M 1;  F 24 | endothelium-dependent and -independent vasodilation | Mean endothelium-dependent vasodilation decrease, but endothelium-independent vasodilation induced by sublingual glycerol triturate did not differ between pSS patients and controls |
| Rachapalli SM. et al, 2009^3^ | UK | case-control; single-center | 8.7±3.2 | pSS(25); controls(25) | 2002 AECG criteria | 61.8±9.1 | M 1;  F 24 | ABI | pSS patients had a higher prevalence of low ABI, but not significant. The subgroup of pSS patients with a longer duration of disease had a significantly lower ABI |
| Gerli R. et al, 2010^4^ | Israel | case-control; single-center | 9.1±6.2 | pSS(45); HC(59) | 2002 AECG criteria | 44±8 | M 0;  F 45 | FMV, NMV | FMV values did not differ, and NMV was lower in SS patients. An NMV decrease was confirmed in SS patient subsets with evidence of leukopenia, rheumatoid factor, anti-SSB antibodies, and joint involvement. |
| Akyel A. et al, 2012^5^ | Turkey | case-control; single-center | NA | pSS(35); HC(20) | 2002 AECG criteria | 47.6 ± 8.0 | M 4;  F 31 | Brachial artery FMD and carotid IMT | Carotid IMT values were similar between groups, brachial artery FMD was disrupted in the pSS group. |
| Atzeni F. et al, 2014^6^ | Italy | case-control; single-center | 3.83±0.69 | pSS(75); controls(68) | 2012 ACR criteria | 60.14±7.81 | M 6;  F 16 | CFR, IMT, PWV, myocardial deformation | Although within the normal range, the patients' CFR was lower. Both left and right PWV values were significantly higher, whereas IMT was substantially similar in the two groups. |
| Cicek OF. et al, 2014^7^ | Turkey | case-control; single-center | NA | SS(50); HC(47) | 2002 AECG criteria | 42.8±8.3 | M 3;  F 47 | Aortic stiffness,  left ventricular systolic and diastolic functions | There is a significant relationship between aortic stiffness and left ventricular diastolic dysfunction in patients with SS in this study. |
| Zardi EM. et al, 2014^8^ | Italy | case-control; single-center | 6.5(median) | pSS(18); mild OA controls（18） | 2012 ACR criteria | 65±5.93 | M 0;  F 18 | IMT, stiffness, hemodynamic parameters | No significant difference was found between pSS and control patients in IMT, stiffness and hemodynamic parameters. |
| Sabio JM. et al, 2015^9^ | Spain | case-control; single-center | 6.0(median) | pSS(44); HC(78) | 2002 AECG criteria | 43(IQR 32–51 years) | M 0;  F 44 | PWV | Women with primary SS had higher PWV, but a similar FRS. The FRS and chronic damage were found to be independently associated with PWV. |
| Gravani F. et al, 2015^10^ | Greece | prospective cohort; single-center | 8.4 ± 7.0 | pSS(64); RA(77); HC(60) | 2002 AECG criteria | 57.2 ± 12.4 | M 4;F 60 | IMT | Almost two thirds of patients with primary SS have subclinical atherosclerosis and impaired bone health, partly attributed to the presence of traditional risk factors as well as disease-related features. |
| Garcia AB. et al, 2016^11^ | Brazil | case series; single-center | 5.78±4.37 | pSS(45) | 2002 AECG criteria | 54.88±12.53 | M 0; F 45 | ABI | A clear correlation between low ABI and the positivity of autoantibodies (antinuclear antibodies, anti-SSA, rheumatoid factor). |
| Sezis Demirci, M. et al, 2016^12^ | Turkey | case-control; single-center | 10(median) | pSS(75); controls(68) | 2002 AECG criteria | 54.0±9.3 | M 0; F 75 | PWV | Mean PWV was higher, and positively correlated with age, BMI, serum cholesterol, LDL, and CRP, blood pressure, MAP, pulse pressure and left ventricular mass index. Arterial stiffness was associated with age, MAP and LDL levels in pSS patients. |
| Zardi EM. et al, 2016^13^ | Italy | case-control; single-center | 8.2(median) | pSS(25); mild OA controls(22) | 2002 AECG criteria | 68±6.5 | M 0; F 25 | IMT | IMT and levels of vitamin D were significantly increased and decreased in pSS, respectively. No correlation was observed between vitamin D and IMT in pSS. Significant positive correlation between disease duration and IMT. |

ABI, ankle brachial index; ACR, American College of Rheumatology; AECG, American-European Consensus Group; BMI, body mass index ; CFR, coronary flow reserve; CRP, C-reactive protein; F, female; FMD, flow mediated dilatation; FRS, Framingham Risk Score; HC, healthy control; IMT, intima-media thickness; IQR, interquartile range; LDL, low-density lipoprotein; M, male; MAP, mean arterial pressure; NA, not available; NMV, nitrate-mediated vasodilation; OA, osteoarthritis; PWV, pulse wave velocity; RA, rheumatoid arthritis; SS, Sjögren's syndrome;

**Table S2** Studies investigating the cardiovascular risk factors in primary Sjögren’s syndrome.

| study | region | study design and setting | study period; mean follow-up years (years) | participants (n) | diagnostic criteria | mean age (years) | sex, n | cardiovascular risk factors | results |
| --- | --- | --- | --- | --- | --- | --- | --- | --- | --- |
| Lodde BM. et al, 2006 | United States | case–control; single-center | 1997- 2003; NA | SS(46); xerostomic controls(12) | 2002 AECG criteria | 53.5±12.3 | M 0;  F 46 | altered lipid levels | HDL and total cholesterol levels were significantly lower in primary SS patients, and in SS patients low total and HDL cholesterol levels were associated with serologically active disease. |
| Gerli R. et al, 2006 | Israel | case–control; single-center | NA; 12 ± 8.4 | SS(37); controls(35) | 2002 AECG criteria | 53.5 ± 12.3 | M 0;  F 37 | dyslipidemia | HDL but not total cholesterol were reduced in pSS. The patients with anti-SSA/SSB antibodies had lower levels of total and HDL cholesterol and increased high-risk HDL cholesterol levels. |
| Ramos-Casals M. et al, 2007 | Spain | case–control; single-center | 1994-2000; 11.32 ± 0.32 | SS(254); general population control (254) | 1993 European Community Study Group diagnostic | 52.35±0.91 | M 20;  F 234 | Serum metabolic alterations  (total cholesterol, triglycerides, glycaemia, and uric acid) | Patients with primary SS showed a higher prevalence of associated dyslipidemia, DM, and hyperuricemia. Metabolic alterations were associated with a differentiated pattern of clinical and immunological SS expression, but not with SS related therapies (except for the higher frequency of DM observed in patients treated with corticosteroids). |
| Cruz W. et al, 2010 | Brazil | case–control; multicenter | NA;  NA | pSS(73); HC(65) | 2002 AECG criteria | 56 ± 13.5 | M 3;  F 70 | abnormal lipoprotein profile | pSS patients frequently present abnormal lipid profile, which are associated with higher levels of ESR. |
| Perez-De-Lis, M. et al, 2010 | Spain | case–control; single-center | 1984-2009; NA | pSS(312); HC(312) | 2002 AECG criteria | NA | NA | traditional CV disease risk factors | Patients with primary SS showed a higher frequency of DM and hypertriglyceridemia and a lower frequency of hypertension and smoking. Corticosteroid use was closely associated with cardiovascular risk factors. |
| Juarez M. et al, 2014 | United Kingdom | Prospective cohort; multicenter | NA;  NA | pSS(543); HC(473) | 2002 AECG criteria | 59.1 ± 12.4 | M 5;  F 538 | traditional CV risk factors | Primary SS patients are more than 2 times more likely to experience hypertension and hypertriglyceridemia |
| Bartoloni, E. et al, 2015 | Italy | retrospective cohort; population-based | NA;  5±6 | pSS(1343);healthy women controls (4774) | 1993 European Community Study Group diagnostic | 57±14 | M 59;  F 1284 | traditional CV disease risk factors | Hypertension and hypercholesterolemia were more prevalent, whereas smoking, obesity and diabetes mellitus were less prevalent, in women with pSS |
| Augusto KL. et al, 2016 | Brazil | case–control; single-center | 2011-2013; NA | pSS(71); female volunteers controls(71) | 2002 AECG criteria | 47.6±10.3 | M 0;  F 71 | Metabolic syndrome | BMI, smoking, sedentariness, and menopause were comparable, whereas MetS, hypertension, and dyslipidemia were more frequent in patients. pSS patients with MetS had higher BMI, waist circumference, cholesterol, LDL-C, triglycerides, insulin, leptin and HOMA-IR values, and greater hypertension and diabetes rates than pSS patients without MetS. |

AECG, American-European Consensus Group; BMI, body mass index; CV, cardiovascular; DM, diabetes mellitus; ESR, erythrocyte sedimentation rate; F, female; HC, healthy control; HDL, high density lipoprotein; HOMA-IR, homeostasis model assessment index; LDL-C, low density lipoprotein-cholesterol M, male; MetS, metabolic syndrome; NA, not available; SS, Sjögren's syndrome;

## Reference

1 Vaudo, G. *et al.* Precocious intima-media thickening in patients with primary Sjogren's syndrome. *Arthritis Rheum* **52**, 3890-3897, doi:10.1002/art.21475 (2005).

2 Pirildar, T. *et al.* Endothelial dysfunction in patients with primary Sjogren's syndrome. *Rheumatology international* **25**, 536-539, doi:10.1007/s00296-005-0599-5 (2005).

3 Rachapalli, S. M., Kiely, P. D. & Bourke, B. E. Prevalence of abnormal ankle brachial index in patients with primary Sjogren's syndrome. *Clinical rheumatology* **28**, 587-590, doi:10.1007/s10067-009-1099-x (2009).

4 Gerli, R. *et al.* Functional impairment of the arterial wall in primary Sjogren's syndrome: combined action of immunologic and inflammatory factors. *Arthritis care & research* **62**, 712-718, doi:10.1002/acr.20117 (2010).

5 Akyel, A. *et al.* Endothelial dysfunction in primary Sjogren syndrome. *The West Indian medical journal* **61**, 870-872 (2012).

6 Atzeni, F. *et al.* New parameters for identifying subclinical atherosclerosis in patients with primary Sjogren's syndrome: a pilot study. *Clinical and experimental rheumatology* **32**, 361-368 (2014).

7 Cicek, O. F. *et al.* Assessment of the relationship between aortic stiffness and left ventricular functions with echocardiography in patients with Sjogren's syndrome. *International journal of rheumatic diseases* **17**, 658-663, doi:10.1111/1756-185x.12258 (2014).

8 Zardi, E. M., Sambataro, G., Basta, F., Margiotta, D. P. & Afeltra, A. M. Subclinical carotid atherosclerosis in elderly patients with primary Sjogren syndrome: a duplex Doppler sonographic study. *International journal of immunopathology and pharmacology* **27**, 645-651, doi:10.1177/039463201402700422 (2014).

9 Sabio, J. M. *et al.* Prevalence of and factors associated with increased arterial stiffness in patients with primary Sjogren's syndrome. *Arthritis care & research* **67**, 554-562, doi:10.1002/acr.22493 (2015).

10 Gravani, F. *et al.* Subclinical atherosclerosis and impaired bone health in patients with primary Sjogren's syndrome: prevalence, clinical and laboratory associations. *Arthritis research & therapy* **17**, 99, doi:10.1186/s13075-015-0613-6 (2015).

11 Garcia, A. B. *et al.* Asymptomatic Atherosclerosis in Primary Sjogren Syndrome: Correlation Between Low Ankle Brachial Index and Autoantibodies Positivity. *Journal of clinical rheumatology : practical reports on rheumatic & musculoskeletal diseases* **22**, 295-298, doi:10.1097/rhu.0000000000000413 (2016).

12 Sezis Demirci, M. *et al.* Is There an Increased Arterial Stiffness in Patients with Primary Sjogren's Syndrome? *Internal medicine (Tokyo, Japan)* **55**, 455-459, doi:10.2169/internalmedicine.55.3472 (2016).

13 Zardi, E. M., Basta, F. & Afeltra, A. Levels of Vitamin D, Disease Activity and Subclinical Atherosclerosis in Post-menopausal Women with Sjogren's Syndrome: Does a Link Exist? *In Vivo* **30**, 721-725 (2016).
